# Global research trends of home pharmaceutical care: a bibliometric analysis via CiteSpace

**DOI:** 10.3389/fmed.2025.1489720

**Published:** 2025-03-28

**Authors:** Qingfang Wu, Xiaorong Feng, Chao Shen, Ying Liu, Shiwen Yang, Na Su

**Affiliations:** ^1^Department of Pharmacy, West China Hospital, Sichuan University, Chengdu, China; ^2^Department of Pharmacy, The First People's Hospital of Shuangliu District, West China (Airport) Hospital of Sichuan University, Chengdu, China; ^3^Department of Pharmacy, Shangjin Hospital, West China Hospital, Sichuan University, Chengdu, China; ^4^West China School of Pharmacy, Sichuan University, Chengdu, China

**Keywords:** bibliometric analysis, CiteSpace, home pharmaceutical care, visualization analysis, family pharmacist

## Abstract

**Background:**

This study aimed to systematically review the research on home pharmaceutical care and to identify emerging trends and research hotspots using bibliometric methods.

**Methods:**

Publications related to home pharmaceutical care, published from inception to 6 February 2025, were extracted from the Web of Science Core Collection (WoSCC). The bibliometric tool CiteSpace was employed to analyze various metrics, including the number of publications, contributing countries, institutions, authors, keywords, cited references, and research trends in the field of home pharmaceutical care.

**Results:**

A total of 812 relevant articles were retrieved from the WoSCC. The most prolific contributors were Hughes CM, Nishtala, PS, and Lapane KL. The United States emerged as the leading country in the field, with Queen’s University Belfast identified as the most productive institution. The keyword with the highest frequency was “pharmaceutical care.” The research hotspots in this field were centered around “polypharmacy,” “medication reconciliation,” and “drug-related problems.”

**Conclusion:**

This study utilized CiteSpace to analyze research trends and hotspots in the field of home pharmaceutical care. The findings suggest that “polypharmacy” and “care homes” are likely to become focal points of future research. Additionally, the development of research in developing countries lags behind that in developed countries. Therefore, it is crucial for developing countries to learn from the advances made by developed nations in this field, and to foster greater international collaboration and research efforts.

## 1 Introduction

Home pharmaceutical care plays a vital role in ensuring the safety and effectiveness of medication management for patients in a home setting. It encompasses several essential functions, including the provision of medications and medical supplies, the establishment and maintenance of comprehensive patient medication profiles, and the facilitation of communication and consultation with other healthcare professionals. Additionally, home pharmaceutical care involves educating patients and caregivers on the correct application and storage of medications, regularly reviewing and evaluating patients’ medication plans, and preventing and monitoring drug-related problems. Pharmacists also provide critical drug information to the healthcare team and offer disease management support when needed. This comprehensive approach is fundamental to optimizing therapeutic outcomes, minimizing risks, and enhancing the overall quality of life for patients receiving care at home (1, 2).

Developed countries, such as the United States and the United Kingdom, were early adopters of home pharmaceutical care services, whereas the development in this area has progressed more slowly in developing countries. The implementation of home pharmaceutical care varies across countries. For instance, in the United States, pharmacists are integrated into the patient-centered medical home (PCMH) model, whereas in China, home pharmaceutical services are provided through collaboration between hospital and community pharmacists. Despite the growing recognition of the importance of home pharmaceutical care, the research hotspots and emerging trends in this field remain somewhat unclear. Bibliometrics, a quantitative statistical analysis tool, is widely used to analyze and track research trends (3–5), while CiteSpace is a software application that facilitates visualization and analysis to reveal research trends and connections among hotspots (6–9). In this study, we reviewed previous research on home pharmaceutical care and conducted an analysis of the research hotspots using bibliometric methods. The findings may help elucidate the historical development of research in this field and suggest new directions for future studies.

## 2 Methods

### 2.1 Search strategy and inclusion criteria

**Figure 1:**
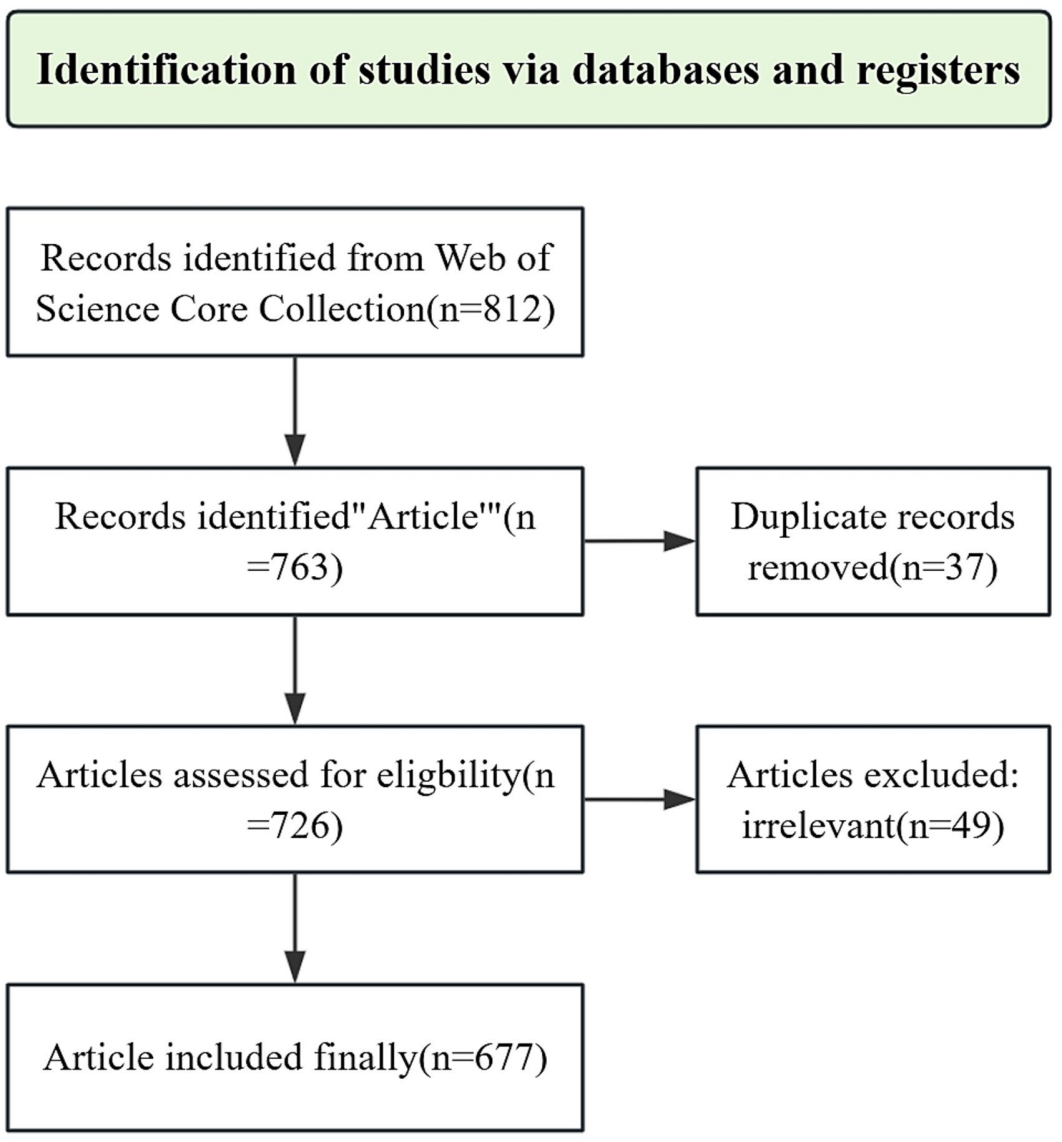
The flowchart of the search strategy.

We performed a comprehensive search of the Web of Science Core Collection (WoSCC), which encompasses the Science Citation Index Expanded (SCI-EXPANDED) and the Social Sciences Citation Index (SSCI), to identify relevant publications from inception through 6 February 2025. The search strategy utilized the term “TS = home pharmaceutical care,” and was limited to articles published in English. To refine the selection, we applied exclusion criteria that eliminated case reports, conference papers, meeting abstracts, duplicate publications, articles lacking keywords or author information, and studies not directly related to the topic (Figure 1).

### 2.2 Bibliometrics and visualization analysis

We exported the selected articles in a plain text format, including full records and references. The data was then imported into CiteSpace 6.3.R1 for conversion and bibliometric analysis. The parameters in CiteSpace were set as follows: time slicing was configured from 1979 to 2025 with three-year intervals per slice; selection criteria were set to the top 10%; and pruning was performed using the pathfinder algorithm, with all other settings left at their default values. Additionally, we utilized CiteSpace to detect and extract keywords and references exhibiting citation bursts to forecast emerging trends in home pharmaceutical care. We used the algorithm of Log Likelihood Ratio (LLR) to clustering keywords and references.

We generated co-citation maps for authors, institutions, countries, keywords, and references, as well as clusters of keywords and references via CiteSpace. In the co-citation maps, different nodes represent various elements (e.g., authors, institutions, references), with the size of each node proportional to its citation count and frequency. Links between nodes indicate mutual citation relationships, while centrality serves as a key indicator of a node’s importance within the field. Nodes with higher centrality values are deemed more influential. The red areas in the tree rings of certain nodes highlight periods of citation or frequency bursts during the field’s development. Different colored points and lines on the map allow us to identify specific years of occurrence or collaboration (10, 11).

To evaluate clustering, we used silhouette (S) values to assess the average contour value of clusters and modularity (Q) values to evaluate the significance of the clustering structure. A silhouette value greater than 0.7 indicates efficient and reliable clusters, while a modularity value exceeding 0.5 signifies a significant cluster structure (12). Additionally, Journal Citation Reports (JCRs) for 2023 were used to obtain journal impact factors. To ensure the accuracy and reliability of the data, two researchers (QW, SZ) independently performed data extraction and analysis management.

No informed consent or ethical approval was required for this study, as all data and information were derived from secondary sources available in the open-access database (WoSCC).

## 3 Results

### 3.1 Article distribution by publication year

**Figure 2:**
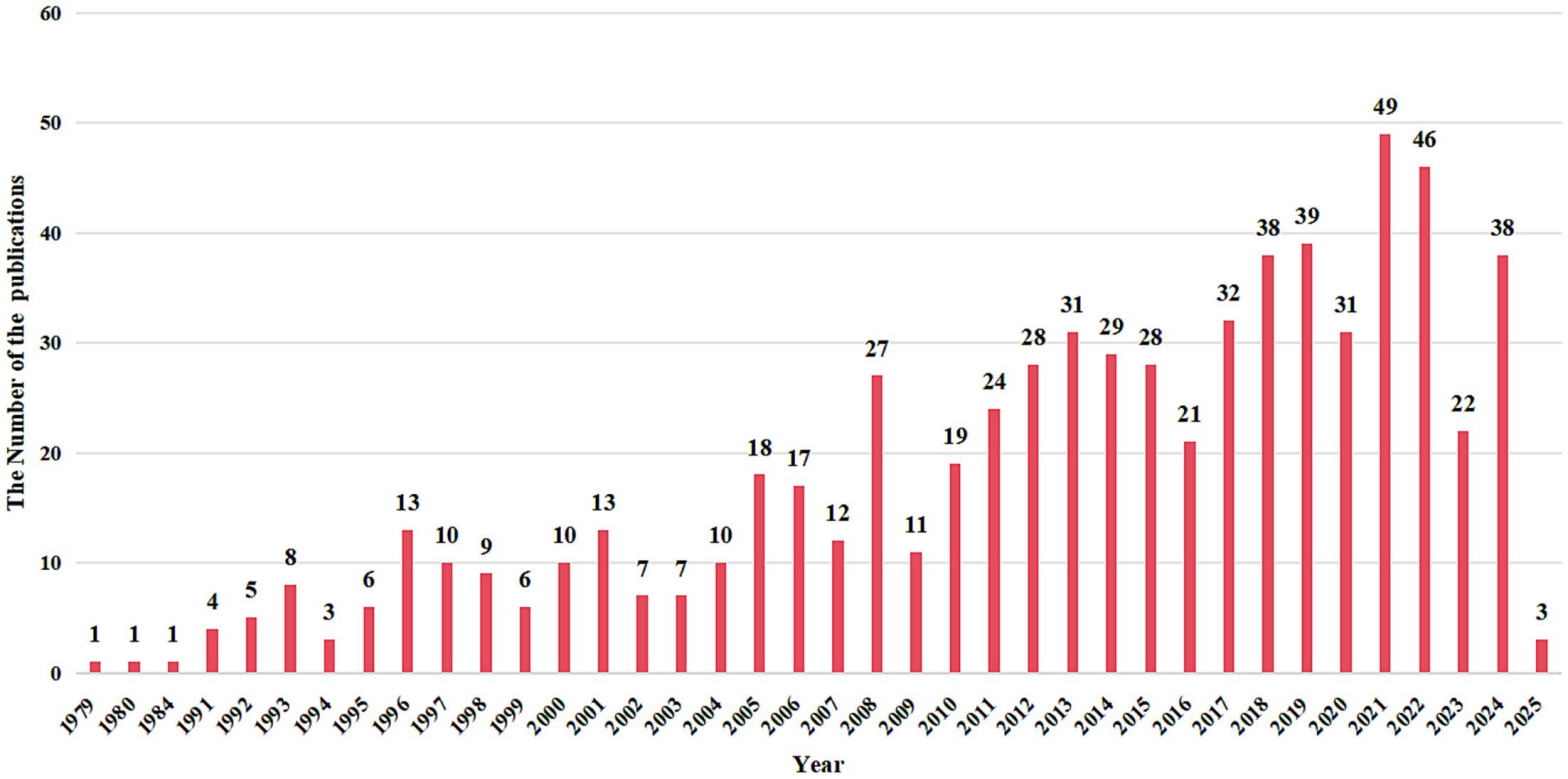
The trend of the number of publications over the years.

After screening 812 publications from the Web of Science Core Collection (WoSCC), we included a total of 677 qualified publications on home pharmaceutical care in our analysis. As illustrated in Figure 2, these articles were published between 1979 and 2025, with an overall upward trend in annual publications over the past 40 years. This consistent growth indicates that the field of home pharmaceutical care holds significant potential for further development.

### 3.2 Cooperation networks among authors, institutions and countries

**Figure 3:**
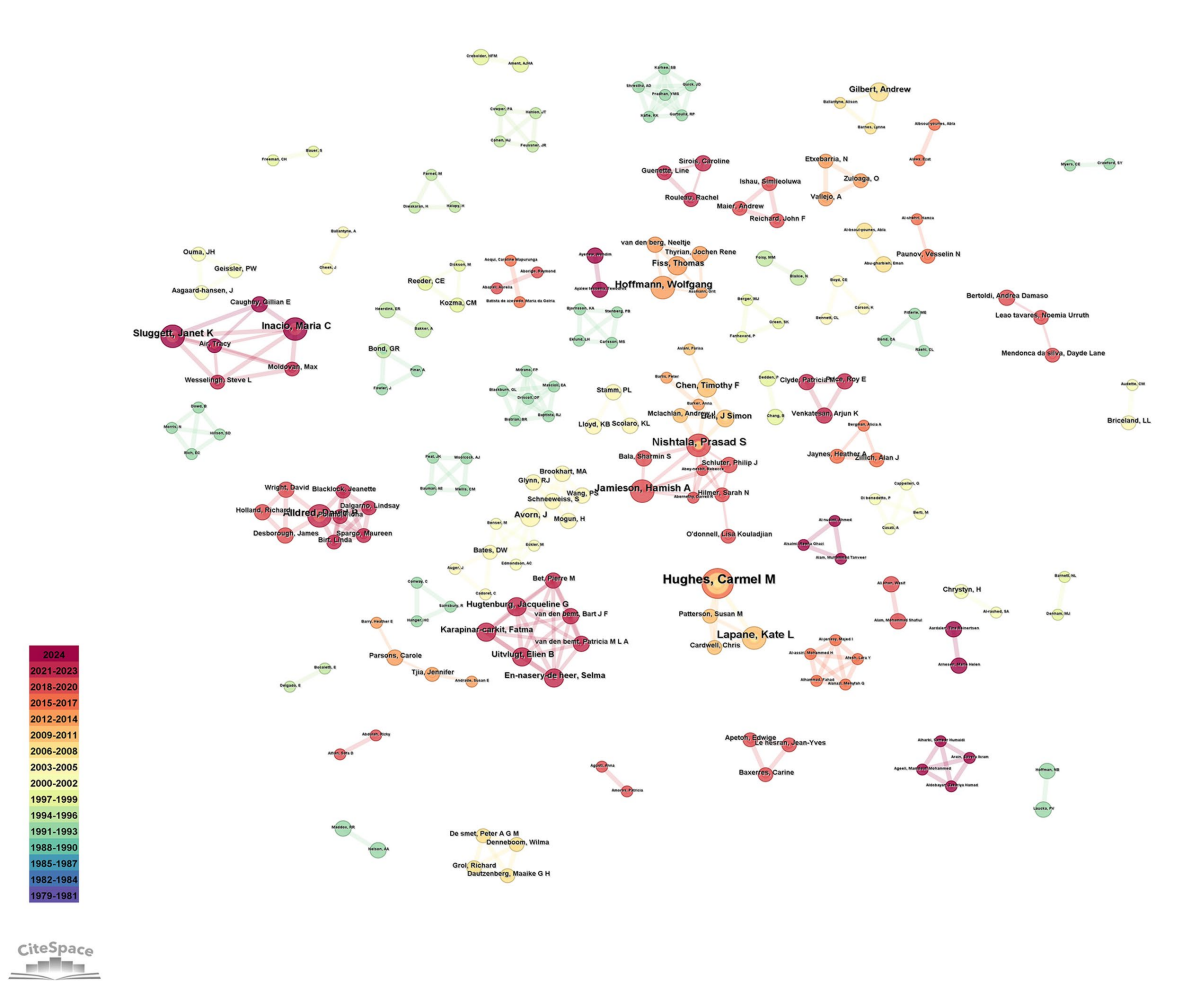
Coauthorship among authors on home pharmaceutical care.

**Figure 4:**
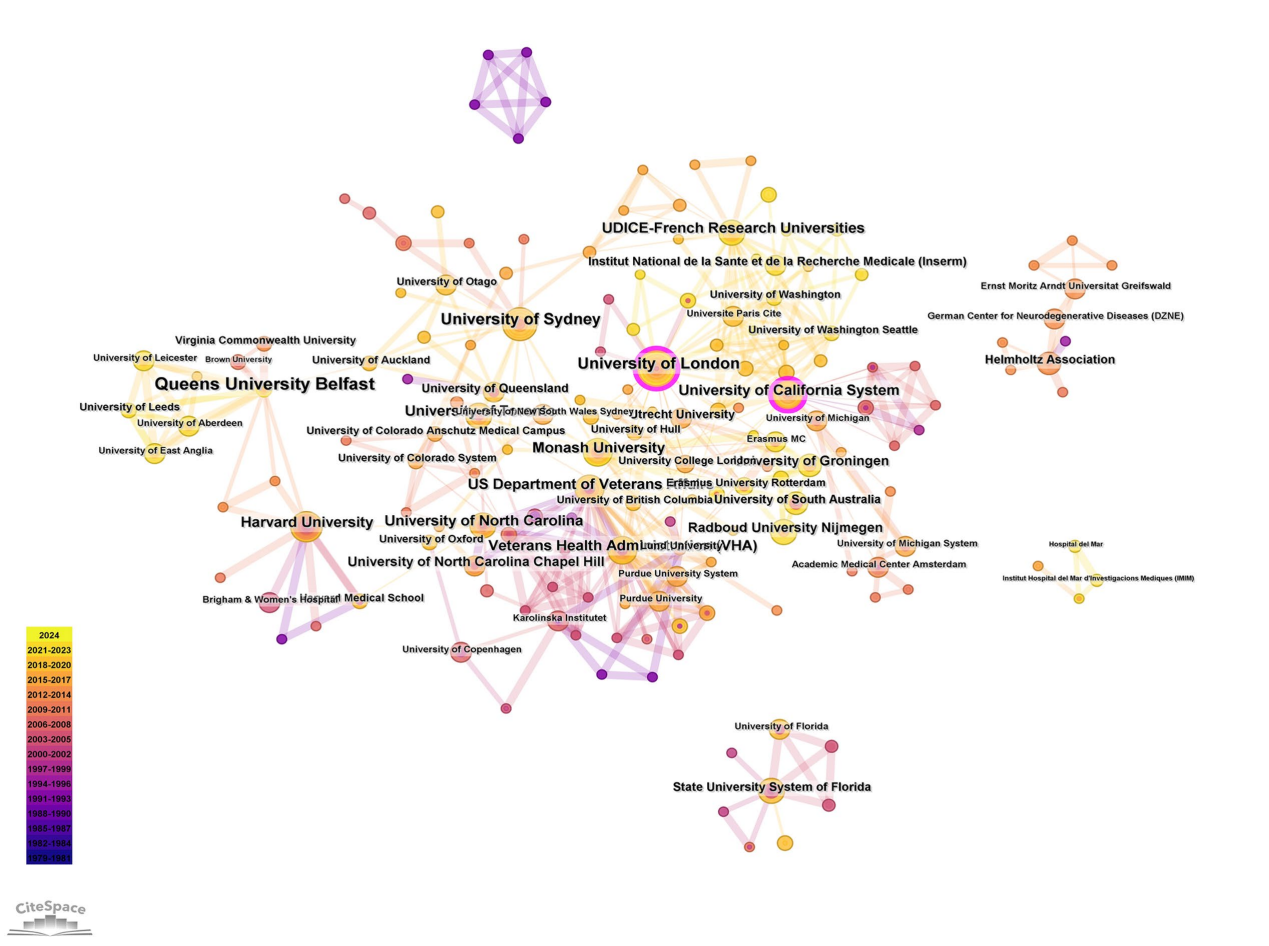
Coauthorship among institutions on home pharmaceutical care.

**Figure 5:**
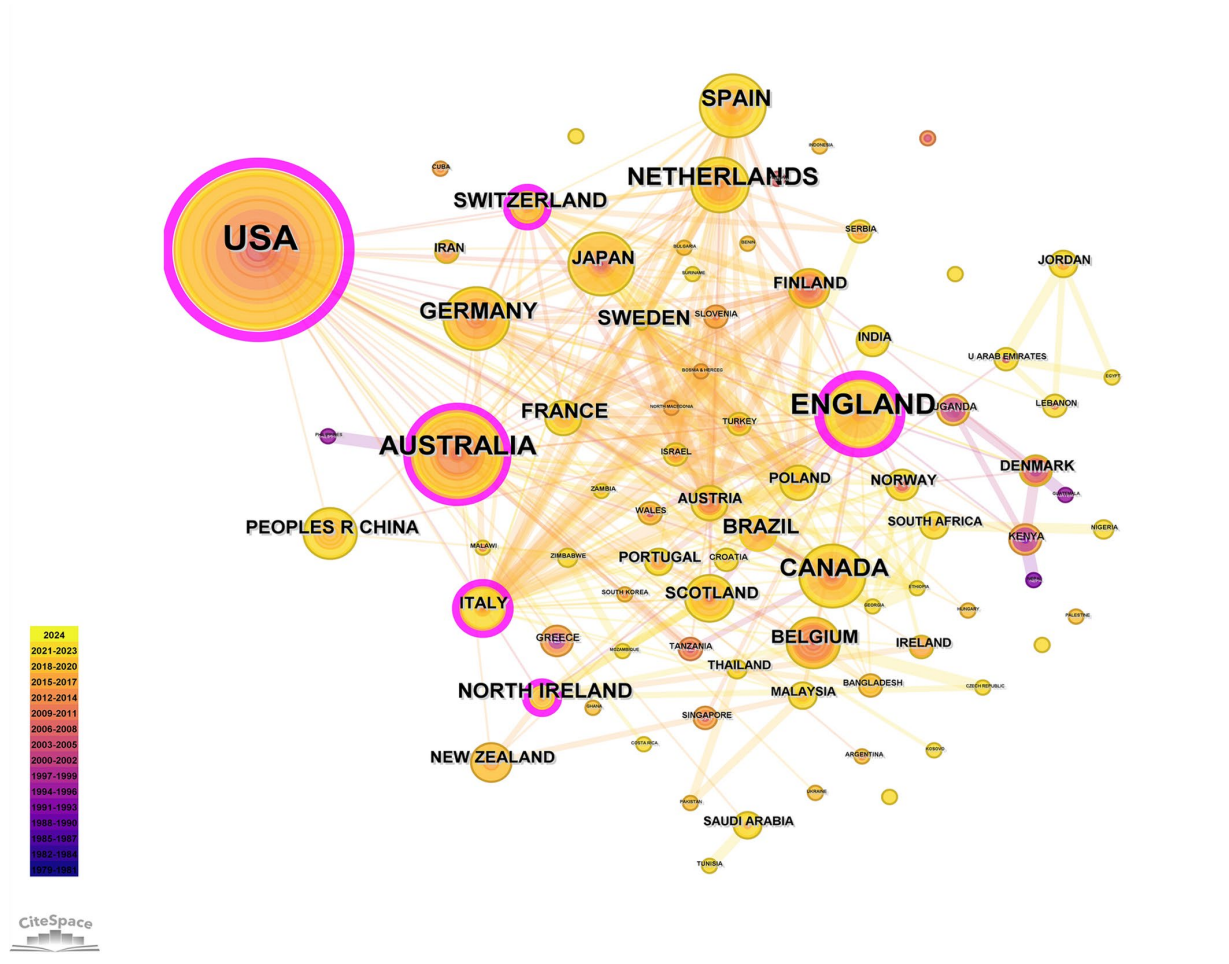
Coauthorship among countries on home pharmaceutical care.

The visualization of the authors’ cooperation network is depicted in Figure 3, with the top three most prolific authors being Hughes, CM (7 articles), Nishtala, PS (5 articles), and Lapane, KL (5 articles). Figure 4 illustrates the distribution of publications by institution, where the Queen’s University Belfast emerges as the most productive, with 18 publications, followed by the University of London (14 articles) and the University of Sydney (13 articles). The cooperation network among countries is presented in Figure 5, highlighting that the United States leads with 200 articles, followed by the United Kingdom with 100 articles, and Australia with 55 articles. Notably, the publication count for the United Kingdom includes contributions from England, Scotland, Northern Ireland, and Wales, as manually calculated (Table 1). The United States demonstrates the highest centrality in the network (centrality = 0.46).

**Table 1:** The top 10 of the most active authors, institutions and countries.

Rank	Categories	Records
Author		
1	Hughes, CM	7
2	Nishtala, PS	5
3	Lapane, KL	5
4	Inacio, MC	4
5	Alldred, DP	4
6	Hoffmann, Wolfgang	4
7	Jamieson, HA	4
8	Sluggett, JK	4
9	Chen, TF	3
10	Uitvlugt, Elien B	3
Institution		
1	Queen’s University Belfast	18
2	The University of London	14
3	The University of Sydney	13
4	Harvard University	12
5	University of California System The University of Queensland	11
6	Veterans Health Administration (VHA)	11
7	Monash University	11
8	US Department of Veterans Affairs	11
9	University of Toronto	10
10	UDICE-French Research Universities	10
Country		
1	USA	200
2	UK	100
3	AUSTRALIA	55
4	NETHERLANDS	42
5	CANADA	36
6	GERMANY	33
7	SPAIN	32
8	BRAZIL	32
9	SWITZERLAND	20
10	CHINA	20

The records of the UK in the article, it should be noted, was the sum of publications of England, Scotland, Northern Ireland and Welsh by manual calculation.

### 3.3 Keywords analysis with cooperation network, clusters, and citation burst

**Figure 6:**
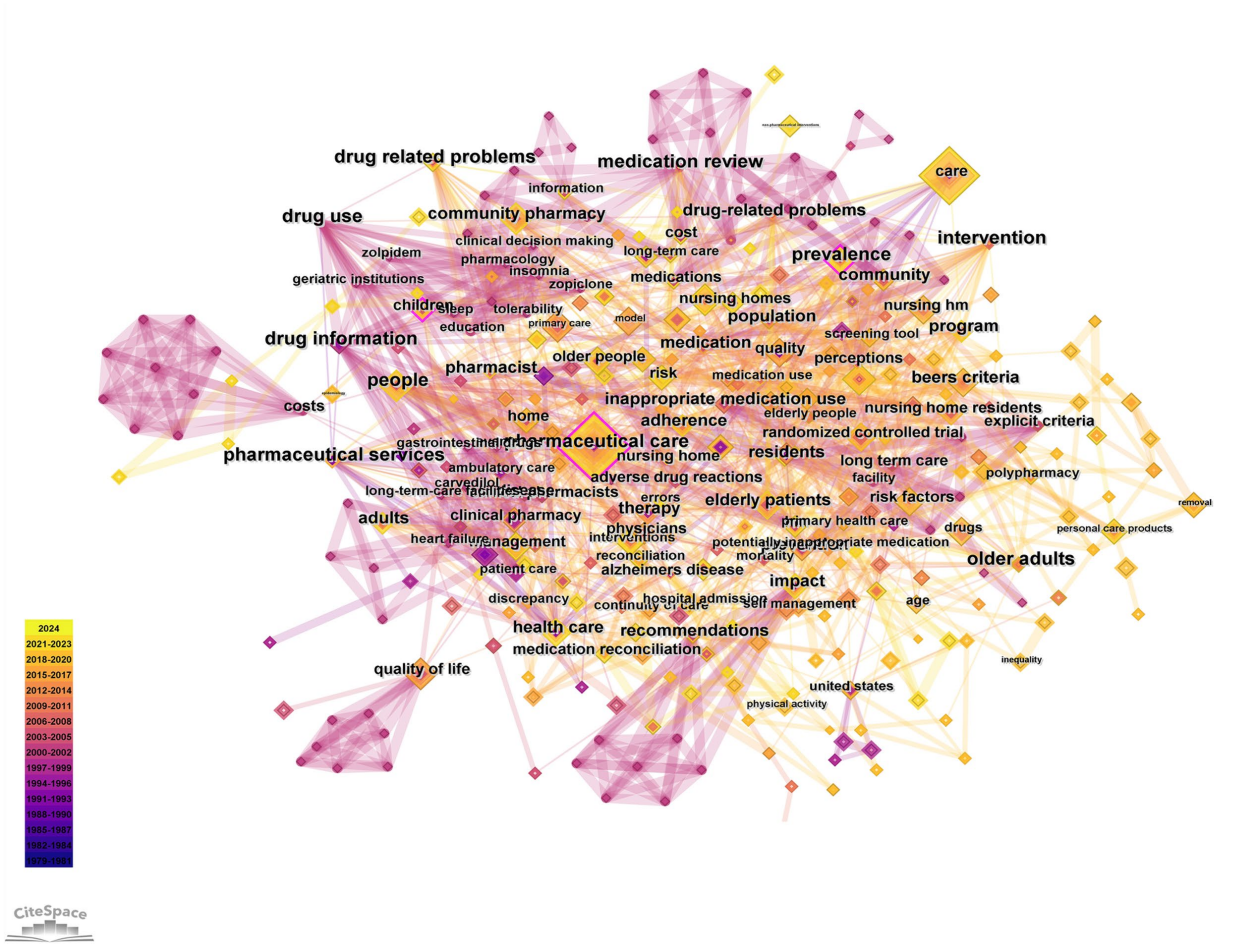
The network of keyword on home pharmaceutical care.

**Figure 7:**
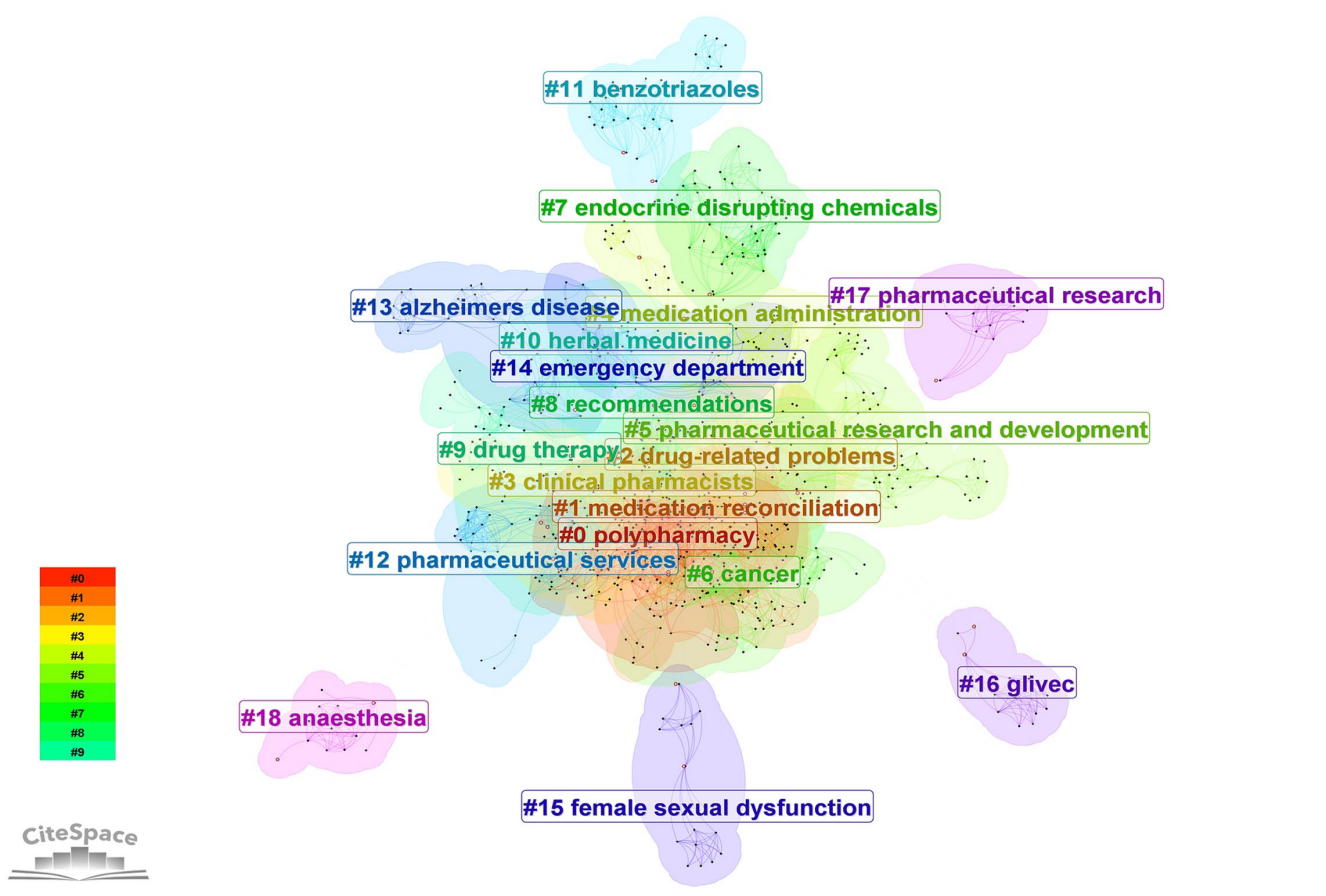
The network of keyword clusters on home pharmaceutical care.

Keyword analysis reveals research hotspots in home pharmaceutical care. Figure 6 shows the keyword network with “pharmaceutical care” (104 records) “care” (81 records), and “management” (45 records) as the top three keywords (Table 2). Clustering identified 19 keyword groups (Figure 7), with the top clusters being #0 polypharmacy, #1 medication reconciliation, #2 drug-related problems. The clustering reliability was high (Q = 0.7035 S = 0.8909). Table 3 lists the top 10 keywords with the strongest citation bursts including “pharmaceutical services” (strength = 10.86, 1992–2008) and “health care” (strength = 7.43, 1992–2005).

**Table 2:** The top 20 keywords in terms of records on home pharmaceutical care research.

Rank	Keywords	Records	Rank	Keywords	Records
1	pharmaceutical care	100	11	outcome	28
2	care	82	12	randomized controlled trial	28
3	management	53	13	health	27
4	pharmaceutical services	44	14	community pharmacy	27
5	impact	42	15	risk	26
6	older people	37	16	quality	26
7	health care	34	17	nursing homes	26
8	prevalence	32	18	adherence	25
9	elderly patient	31	19	home	23
10	people	30	20	program	23

**Table 3:** Top 10 keywords with the strongest citation bursts.

Keywords	Year	Strength	Begin	End	1979–2025
pharmaceutical services	1992	10.86	1992	2008	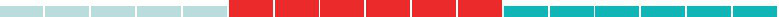
health care	1992	7.43	1992	2005	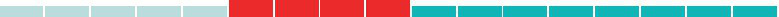
data collection	1992	5.14	1992	1999	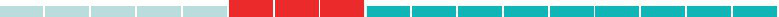
ambulatory care	1996	4.82	1996	2008	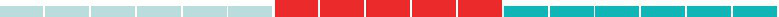
mortality	2003	5.25	2003	2011	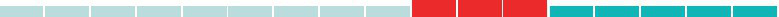
elderly patients	2003	4.62	2003	2011	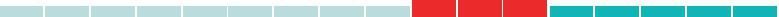
primary care	2007	4.44	2007	2017	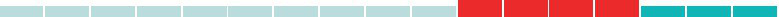
randomized controlled trial	2007	4.27	2007	2011	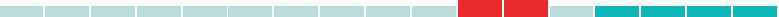
quality of life	2000	3.9	2012	2017	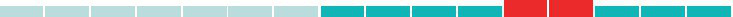
non-pharmaceutical interventions	2021	3.9	2021	2022	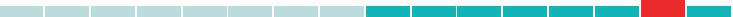

### 3.4 Co-citation analysis on authors, journals

Supplementary Figures 1, 2 display author and journal co-citation networks. The World Health Organization (WHO) was the most cited author (86 records). Among journals, Journal of the American Geriatrics Society (IF2023 = 4.3, 170 records) ranked first, followed by Journal of the American Medical Association (JAMA) (IF2023 = 63.1, 150 records) and PLOS ONE (IF2023 = 2.9, 147 records) (Supplementary Table 1).

### 3.5 References analysis with co-citation, clusters, and citation burst

Visualization analysis of references can also aid in identifying key research hotspots within this field. As illustrated in Supplementary Figure 3, the top three most co-cited references are authored by Alldred DP (2016), Moher D (2015), and Masnoon N (2017). We conducted cluster analysis (Supplementary Figure 4) and burst analysis (Table 4) on the cited literature, with the article by Alldred DP exhibiting the highest burst strength of 4.97.The most frequent co-cited reference (written by Alldred DP in 2016) was about that pharmacist-led interventions to optimize prescribing for elders in care homes had some function of identifying and solving some drug related problems, as well as improving drug appropriateness (13). There were three references (written by Masnoon N in 2017, Schluter PJ in 2016, Abbott RA in 2020) were the research hotspots with high citation burst in recent years (Table 4), which mainly researched into polypharmacy, COVID-19 and old people (14–16).

**Table 4:** Top 10 references with the strongest citation bursts.

References	Year	Strength	Begin	End	1979–2025
Holland R, 2008, BRIT J CLIN PHARMACO, V65, P303, DOI 10.1111/j.1365-2125.2007.03071.x	2008	3.56	2011	2013	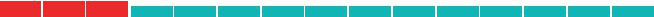
Forsetlund L, 2011, BMC GERIATR, V11, P0, DOI 10.1186/1471-2318-11-16	2011	3.52	2011	2016	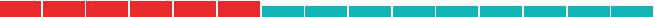
Kaur S, 2009, DRUG AGING, V26, P1013, DOI 10.2165/11318890-000000000-00000	2009	3.11	2011	2013	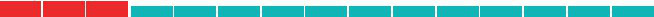
Gillespie U, 2009, ARCH INTERN MED, V169, P894, DOI 10.1001/archinternmed.2009.71	2009	3.11	2011	2013	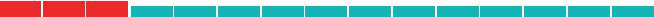
Moher D, 2015, SYST REV-LONDON, V4, P0, DOI 10.1186/2046-4053-4-1	2015	3.82	2015	2019	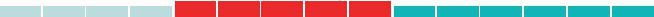
Alldred DP, 2013, COCHRANE DB SYST REV, V0, P0, DOI 10.1002/14651858.CD009095.pub2	2013	3.04	2014	2019	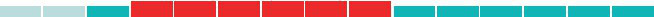
Alldred DP, 2016, COCHRANE DB SYST REV, V0, P0, DOI 10.1002/14651858.CD009095.pub3	2016	4.97	2017	2022	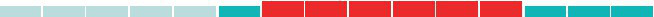
Frankenthal D, 2014, J AM GERIATR SOC, V62, P1658, DOI 10.1111/jgs.12993	2014	3.23	2017	2019	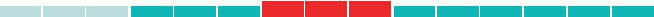
Masnoon N, 2017, BMC GERIATR, V17, P0, DOI 10.1186/s12877-017-0621-2	2017	3.27	2020	2022	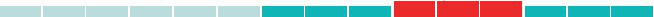
Fick DM, 2019, J AM GERIATR SOC, V67, P674, DOI 10.1111/jgs.15767	2019	3.09	2020	2025	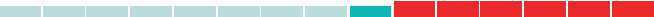

## 4 Discussion

### 4.1 Current status of the development of home pharmacy services

Over the past four decades, a growing body of research has focused on home pharmaceutical care, leading to a surge of contributions in this emerging field. Nine of the top 10 most productive countries were developed nations, with the United States demonstrating the strongest scholarly influence (centrality = 0.46). These findings underscore significant disparities in the advancement of home pharmaceutical care services between developed and developing economies. Family pharmacist services in foreign countries are mainly undertaken by community pharmacists, providing patients with a variety of medical and health-related items of pharmacy services, such as anticoagulation management, pain management, body quality management, drug counseling and so on, and a lot of studies have found that community pharmacists through home pharmaceutical services can improve patients’ medication adherence and therapeutic effects, reduce the expenditure of drug costs, and reduce the readmission rate of discharged patients, and so on. In UK, Pharmacists conduct home visits within 3 days of discharge to optimize medication use, collaborating with GPs in team meetings to develop personalized treatment plans. This model reduces hospital readmissions by 22% through improved medication adherence (NHS England, 2023). Some developing countries, on the other hand, are following the example of developed countries (e.g., conducting contracted teams of family pharmacists), but have not developed a system. But it’s worth mentioning, in recent years, several studies have shown that home pharmaceutical care can significantly reduce the number of medications and interactions in patients with overuse of multiple medications and high healthcare utilization in the developing countries (17).

Bibliometric analyses reveal the following key factors contributing to lagging development in developing countries:

a Late Initiation of Services

The earliest literature on home pharmaceutical care dates to 1979 (United States), whereas developing countries, such as China (the highest contributor in publication volume), published their first research only in 2011.

b Deficiencies in Pharmacist Training Systems

Developing countries lack standardized training frameworks comparable to those in developed nations. Most community pharmacists in these regions cannot independently deliver home pharmaceutical care. For instance: Training systems in developing countries prioritize complex clinical education. In contrast, U.S. institutions implement experiential residency programs where pharmacy students lead transitional care for inpatients and outpatients, including medication reconciliation, discharge counseling, and post-discharge follow-ups. Such training equips graduates with patient communication skills and medication management expertise (18).

c Inadequate Compensation Mechanisms

Community pharmacists in developed nations receive remuneration through government subsidies (e.g., NHS funding), medicare payment system, or third-party platforms. Conversely, most developing countries do not charge for home pharmaceutical services, resulting in limited financial incentives for pharmacists to transition into this field.

d Shortage of Pharmacists

The scarcity of qualified professionals further exacerbates disparities. In China, for example, as of April 2016, China had 413,774 licensed pharmacists, with only 277,967 registered practitioners—a pharmacist-to-population ratio of 1:4,643. This ratio is substantially lower than in developed nations (e.g., the ratio of USA is 1:1,200), and fewer than 15% of licensed pharmacists practice in community settings, where demand for home pharmaceutical services is highest.

In conclusion, the combined effects of delayed initiation, insufficient training, inadequate incentives, and workforce shortages have resulted in marked gaps in both the quantity and quality of home pharmaceutical care services between developing and developed economies. Addressing these systemic challenges requires policy reforms, standardized training frameworks, and sustainable reimbursement models to align with global best practices.

### 4.2 Research hotspots

Based on the visualization results by CiteSpace, we summarized the hot research on home pharmaceutical care.

#### 4.2.1 Medication reconciliation

Medication reconciliation aims to ensure patient medical safety to the maximum extent, achieve the accuracy and continuity of drug treatment, and reduce clinical medication errors and adverse drug events (ADEs). An important task of home pharmaceutical care is medication reconciliation to prevent harm from medications as much as possible. Many scholars have demonstrated that implementing medication reconciliation by pharmacists at all medical care periods is an effective strategy for preventing adverse drug events (19–21). Medication reconciliation participated by pharmacists in primary health care indicates to be efficacious to reduce the number of patients with potentially inappropriate medications (PIMs), which might improve the quality of pharmacotherapy in old people (22). As in many sectors of healthcare, pharmacists play important roles in the home pharmaceutical care of discharged patients. Completion of medication reconciliation by community pharmacists and primary care pharmacists has been prioritized (23). Pharmacists help patients to reduce the readmission rate and medical costs of health care by effective medication reconciliation (24, 25). Michaelsen et al. (26) conducted a retrospective study of differences in medication continuation after discharge and found that 20%~87% of patients were of vary difference in medication use after discharge and the most common reasons are medication omissions and differences in administration method/frequency/dose. It is apparently essential to carry out home pharmaceutical care for discharged patients to ensure the effectiveness and safety of patient medication.

#### 4.2.2 Polypharmacy

Polypharmacy is usually defined as the simultaneous medication therapy with five or more drugs. Polypharmacy is associated with multiple adverse outcomes, including mortality, falls, adverse drug reactions, prolonged hospitalization, and readmission. Natali et al. (27) revealed a high prevalence of polypharmacy in long-term care facilities, with up to 91%, 74%, and 65% of residents taking more than 5, 9, and 10 medications, respectively. Polypharmacy is common in older populations with multiple diseases, accompanied by the use of one or more drugs to treat each disease. Leelakanok et al. (28) found that multidrug therapy is associated with an increase in mortality, and proposed the necessity of achieving the best balance between risk and benefit in healthcare through drug prescription. Compared with usual care, home pharmaceutical care for polypharmacy patients reduced the probability of receiving ≥10 medications and the mean number of PIMs (29). Greater professional autonomy medication review can optimize pharmaceutical care. As the role of home pharmaceutical care is expanding in many countries, this role shows what more could be achieved with pharmacists.

#### 4.2.3 Special populations

Some special populations, such as elderly patients, women and persons with multiple chronic diseases, are at higher risk of PIMs (30). These special patients require more home pharmaceutical care. PIM is an important public health problem, particularly among older people over 65 years (31). Wimmer et al. (32) found that the increased hospitalization rates are closely related to the worse medication adherence of patients and complexity of medication regimens. Another study found that elderly patients with less complex medication regimens may voluntarily discontinue medication when they feel no improvement in their self-perception. Jerry et al. discovered, by monitoring 18 participating sanatoriums and almost 30,000 individuals during the observation period, a great number of ADEs and PIMs emerged. Some errors can be prevented during the ordering stage and errors during monitoring stages are the most common. The other errors in transcription, allocation, and administration are relatively rare to find. Psychoactive drugs and anticoagulants are the most common drugs associated with preventable adverse drug events (33). Alqenae et al. (34) found that adverse drug events after discharge have a significant potential risk. The most common factor is organizational coordination among workers in various functions. The 2019 American Geriatrics Society Beers Criteria lists PIMs to be avoided in older adults (35). The criteria’s new list released by this standard emphasizes the importance of selecting drugs based on patients’ renal function, including drugs that should be avoided or drugs that require dosage adjustments, as well as selecting drugs with no interactions documented to be associated with harms in older adults (36).

### 4.3 Findings and limitations

This study was the first to use CiteSpace to analyze the research hotspots of home pharmaceutical care. We found that over time, research hotspots in this field have evolved from pharmaceutical services to specific medication reconciliation, polypharmacy, disease management for special populations, and so on. The progress of home pharmacy services in developing countries is relatively slow, but the development content and direction are basically similar to those in developed countries. It should be noted that, due to the spread of the COVID-19 epidemic, “personal care product” and “nonpharmaceutical intervention” emerged as new research hotspots in recent years (37–40).

A few limitations of this visualization analysis must be considered. All of our publications came from the Web of Science (WoS) Core Collection database, and articles published in other ways were excluded. Our findings in this study may not be comprehensive because of limited literature. Particularly, this may lead to a certain lag in our analysis of the development of family pharmacy services in non-English speaking countries. Secondly, the CiteSpace software does not clearly distinguish the first author from the corresponding author. Thirdly, the top 50 authors, countries, institutions, and keywords were selected as the selection strategy, which may have some bias. However, we argue that this study can be used to describe the hotspots and emerging trends in this field.

## 5 Conclusion

We have found that the hotspots of home pharmaceutical care are concentrated in home pharmaceutical care and community pharmacy. Community pharmacists are the main body in providing home pharmacy services. Early research in this field mainly focused on developed countries such as the United States, the United Kingdom and Australia. Foreign countries have already reserved and established sufficient talent teams. In recent years, developing countries such as China have also actively carried out related research, and we still need to continue our efforts.

## Data Availability

The original contributions presented in the study are included in the article/Supplementary material, further inquiries can be directed to the corresponding author.

## References

[1] Torrico S. App takes the lead in home health care. Am Pharm. (1979) 19:50–1. doi: 10.1016/S0160-3450(15)32232-7

[2] Olesen C Harbig P Buus KM Barat I Damsgaard EM. Impact of pharmaceutical care on adherence, hospitalisations and mortality in elderly patients. Int J Clin Pharm. (2014) 36:163–71. doi: 10.1007/s11096-013-9898-1, PMID: 24293339

[3] Akmal M Hasnain N Rehan A Iqbal U Hashmi S Fatima K . Glioblastome multiforme: a bibliometric analysis. World Neurosurg. (2020) 136:270–82. doi: 10.1016/j.wneu.2020.01.027, PMID: 31953095

[4] von Berlepsch D Lemke F Gorton M. The importance of corporate reputation for sustainable supply chains: a systematic literature review, bibliometric mapping, and research agenda. J Bus Ethics. (2022) 189:1–26. doi: 10.1007/s10551-022-05268-x, PMID: 36259069 PMC9559133

[5] Železnik D Blažun Vošner H Kokol PA. Bibliometric analysis of the journal of advanced nursing, 1976-2015. J Adv Nurs. (2017) 73:2407–19. doi: 10.1111/jan.13296 28295539

[6] Chen C. A glimpse of the first eight months of the covid-19 literature on microsoft academic graph: themes, citation contexts, and uncertainties. Front Res Metr Anal. (2020) 5:607286. doi: 10.3389/frma.2020.607286, PMID: 33870064 PMC8025977

[7] Liang YD Li Y Zhao J Wang XY Zhu HZ Chen XH. Study of acupuncture for low back pain in recent 20 years: a bibliometric analysis via citespace. J Pain Res. (2017) 10:951–64. doi: 10.2147/JPR.S132808, PMID: 28479858 PMC5411170

[8] Lin X Yang Q Zheng D Tian H Chen L Wu J . Scientometric analysis of lipid metabolism in breast neoplasm: 2012-2021. Front Physiol. (2023) 14:1042603. doi: 10.3389/fphys.2023.1042603, PMID: 37179822 PMC10168182

[9] Miao Y Liu R Pu Y Yin L. Trends in esophageal and esophagogastric junction cancer research from 2007 to 2016: a bibliometric analysis. Medicine. (2017) 96:e6924. doi: 10.1097/MD.0000000000006924, PMID: 28514311 PMC5440148

[10] Chen C. Science mapping: a systematic review of the literature. J Data Informat Sci. (2017) 2:1–40. doi: 10.1515/jdis-2017-0006

[11] Yin M Wang H Sun Y Xu C Ye J Ma J . Global trends of researches on lumbar spinal stenosis: a bibliometric and visualization study. Clin Spine Surg. (2022) 35:E259–e266. doi: 10.1097/BSD.0000000000001160, PMID: 33769984

[12] Zhang L Mei S Zhu B Zhao Z. Trends in research on acute lung injury/acute respiratory distress syndrome associated with viral pneumonia from 1992 to 2022: a 31-year bibliometric analysis. Front Med. (2023) 10:1158519. doi: 10.3389/fmed.2023.1158519, PMID: 37359015 PMC10288490

[13] Alldred DP Kennedy MC Hughes C Chen TF Miller P. Interventions to optimise prescribing for older people in care homes. Cochrane Database Syst Rev. (2016) 15:255–9. doi: 10.1002/14651858, PMID: 26866421 PMC7111425

[14] Masnoon N Shakib S Kalisch-Ellett L Caughey GE. What is polypharmacy? A systematic review of definitions. BMC Geriatr. (2017) 17:230. doi: 10.1186/s12877-017-0621-2, PMID: 29017448 PMC5635569

[15] Schluter PJ Ahuriri-Driscoll A Anderson TJ Beere P Brown J Dalrymple-Alford J . Comprehensive clinical assessment of home-based older persons within New Zealand: an epidemiological profile of a national cross-section. Aust N Z J Public Health. (2016) 40:349–55. doi: 10.1111/1753-6405.12525, PMID: 27197797

[16] Abbott RA Moore DA Rogers M Bethel A Stein K Coon JT. Effectiveness of pharmacist home visits for individuals at risk of medication-related problems: a systematic review and meta-analysis of randomised controlled trials. BMC Health Serv Res. (2020) 20:39. doi: 10.1186/s12913-019-4728-3, PMID: 31941489 PMC6961241

[17] Wang TC Trezise D Ku PJ Lu HL Hsu KC Hsu PC. Effect of pharmacist intervention on a population in Taiwan with high healthcare utilization and excessive polypharmacy. Int J Environ Res Public Health. (2019) 16:2208. doi: 10.3390/ijerph16122208, PMID: 31234455 PMC6617324

[18] Newsom LC Dupree LH Thurston MM Liaot V Nwaesei AS. A scoping review of student pharmacist-led transitions-of-careinitiatives. Am J Pharm Educ. (2023) 87:100001. doi: 10.1016/j.ajpe.2023.02.001, PMID: 37316136

[19] Mekonnen AB McLachlan AJ Brien JA. Effectiveness of pharmacist-led medication reconciliation programmes on clinical outcomes at hospital transitions: a systematic review and meta-analysis. BMJ Open. (2016) 6:e010003. doi: 10.1136/bmjopen-2015-010003, PMID: 26908524 PMC4769405

[20] Institute for Healthcare Improvement. How-to guide: Prevent adverse drug events by implementing medication reconciliation. Cambridge, Massachusetts, USA: Institute for Healthcare Improvement (2011).

[21] Al-Hashar A Al-Zakwani I Eriksson T Sarakbi A Al-Zadjali B Al MS . Impact of medication reconciliation and review and counselling, on adverse drug events and healthcare resource use. Int J Clin Pharm. (2018) 40:1154–64. doi: 10.1007/s11096-018-0650-8, PMID: 29754251

[22] Milos V Rekman E Bondesson Å Eriksson T Jakobsson U Westerlund T . Improving the quality of pharmacotherapy in elderly primary care patients through medication reviews: a randomised controlled study. Drugs Aging. (2013) 30:235–46. doi: 10.1007/s40266-013-0057-0, PMID: 23408163

[23] McNab D Bowie P Ross A MacWalter G Ryan M Morrison J. Systematic review and meta-analysis of the effectiveness of pharmacist-led medication reconciliation in the community after hospital discharge. BMJ Qual Saf. (2018) 27:308–20. doi: 10.1136/bmjqs-2017-007087, PMID: 29248878 PMC5867444

[24] Lehnbom EC Stewart MJ Manias E Westbrook JI. Impact of medication reconciliation and review on clinical outcomes. Ann Pharmacother. (2014) 48:1298–312. doi: 10.1177/1060028014543485, PMID: 25048794

[25] Polinski JM Moore JM Kyrychenko P Gagnon M Matlin OS Fredell JW . An insurer's care transition program emphasizes medication reconciliation, reduces readmissions and costs. Health Aff. (2016) 35:1222–9. doi: 10.1377/hlthaff.2015.0648, PMID: 27385237

[26] Michaelsen MH McCague P Bradley CP Sahm LJ. Medication reconciliation at discharge from hospital: a systematic review of the quantitative literature. Pharmacy. (2015) 3:53–71. doi: 10.3390/pharmacy3020053, PMID: 28975903 PMC5597088

[27] Jokanovic N Tan EC Dooley MJ Kirkpatrick CM Bell JS. Prevalence and factors associated with polypharmacy in long-term care facilities: a systematic review. J Am Med Dir Assoc. (2015) 16:535.e1–535.e12. doi: 10.1016/j.jamda.2015.03.003, PMID: 25869992

[28] Leelakanok N Holcombe AL Lund BC Gu X Schweizer ML. Association between polypharmacy and death: a systematic review and meta-analysis. J Am Pharm Assoc. (2017) 57:729–38. doi: 10.1016/j.japh.2017.06.002 28784299

[29] Garland CT Guénette L Kröger E Carmichael PH Rouleau R Sirois C. A new care model reduces polypharmacy and potentially inappropriate medications in long-term care. J Am Med Dir Assoc. (2021) 22:141–7. doi: 10.1016/j.jamda.2020.09.039, PMID: 33221164

[30] Nothelle SK Sharma R Oakes A Jackson M Segal JB. Factors associated with potentially inappropriate medication use in community-dwelling older adults in the United States: a systematic review. Int J Pharm Pract. (2019) 27:408–23. doi: 10.1111/ijpp.12541, PMID: 30964225 PMC7938818

[31] Patterson SM Cadogan CA Kerse N Cardwell CR Bradley MC Ryan C . Interventions to improve the appropriate use of polypharmacy for older people. Cochrane Database Syst Rev. (2018) 9:Cd008165. doi: 10.1002/14651858.CD008165.pub3, PMID: 30175841 PMC6513645

[32] Wimmer BC Cross AJ Jokanovic N Wiese MD George J Johnell K . Clinical outcomes associated with medication regimen complexity in older people: a systematic review. J Am Geriatr Soc. (2017) 65:747–53. doi: 10.1111/jgs.14682, PMID: 27991653

[33] Gurwitz JH Field TS Avorn J McCormick D Jain S Eckler M. Incidence and preventability of adverse drug events in nursing homes. Am J Med. (2000) 109:87–94. doi: 10.1016/s0002-9343(00)00451-4, PMID: 10967148

[34] Alqenae FA Steinke D Carson-Stevens A Keers RN. Analysis of the nature and contributory factors of medication safety incidents following hospital discharge using national reporting and learning system (nrls) data from England and wales: a multi-method study. Ther Adv Drug Saf. (2023) 14:20420986231154365. doi: 10.1177/20420986231154365, PMID: 36949766 PMC10026140

[35] American Geriatrics Society 2015 Beers Criteria Update Expert Panel. American geriatrics society 63 updated beers criteria for potentially inappropriate medication use in older adults. J Am Geriatr Soc. (2015) 71:2227–46. doi: 10.1111/jgs.13702, PMID: 26446832

[36] Park JW Roh JL Lee SW Kim SB Choi SH Nam SY . Effect of polypharmacy and potentially inappropriate medications on treatment and posttreatment courses in elderly patients with head and neck cancer. J Cancer Res Clin Oncol. (2016) 142:1031–40. doi: 10.1007/s00432-015-2108-x, PMID: 26744323 PMC11819017

[37] Banholzer N Lison A Özcelik D Stadler T Feuerriegel S Vach W. The methodologies to assess the effectiveness of non-pharmaceutical interventions during covid-19: a systematic review. Eur J Epidemiol. (2022) 37:1003–24. doi: 10.1007/s10654-022-00908-y, PMID: 36152133 PMC9510554

[38] Diarra M Kebir A Talla C Barry A Faye J Louati D . Non-pharmaceutical interventions and covid-19 vaccination strategies in Senegal: a modelling study. BMJ Glob Health. (2022) 7:e007236. doi: 10.1136/bmjgh-2021-007236, PMID: 35193893 PMC8882665

[39] Deierlein AL Grayon AR Zhu X Sun Y Liu X Kohlasch K . Personal care and household cleaning product use among pregnant women and new mothers during the covid-19 pandemic. Int J Environ Res Public Health. (2022) 19:5645. doi: 10.3390/ijerph19095645, PMID: 35565038 PMC9104147

[40] Nasr ZG Elamin W Basil M Eljaaly K. Pharmacist-driven antimicrobial stewardship interventions in patients with covid-19: a scoping review. Int J Clin Pharm. (2023) 45:613–21. doi: 10.1007/s11096-023-01574-0, PMID: 37162655 PMC10171144

