# Supplementary material for: Global research trends of home pharmaceutical care: a bibliometric analysis via CiteSpace
Source: Front Med (Lausanne). 2025 Mar 28;12:1489720. doi: 10.3389/fmed.2025.1489720 (PMC11985810; doi:10.3389/fmed.2025.1489720)
Supplement: Supplementary file 1 [file Data_Sheet_1.pdf]

Supplementary Material

Global research trends of home pharmaceutical care: A bibliometric analysis via CiteSpace

Qingfang Wu 1,4†, Xiaorong Feng 1,2†, Chao Shen 1†，Ying Liu 1, Shiwen Yang 1, Na Su 1, 3*

*** Correspondence:** Na Su: zoya159@163.com.

Supplementary Table 1. The top 10 co-cited journals and authors with the highest citations

Rank	Journals	IF (2023)	JCR	Citations	Authors	Citations
1	Journal of the American Geriatrics Society	4.3	Q1	170	WORLD HEALTHORGANIZATION	54
2	the JAMA (Journal of the American Medical Association)	63.1	Q1	150	ZERMANSKY AG	39
3	PLOS ONEARCHIVES OF INTERNAL MEDICINE	2.9	Q1	147	SPINEWINE A	36
4	ARCH INTERN MED	N/A	N/A	146	HOLLAND R	34
5	Bmj-British Medical Journal	133.1	Q1	138	ALLDRED DP	32
6	AGE AND AGEING	6.0	Q1	133	PATTERSON SM	32
7	LANCET	59.1	Q1	129	GALLAGHER P	31
8	British Journal of Clinical Pharmacology	3.1	Q2	127	FURNISS L	29
9	New England Journal of Medicine	96.2	Q1	126	HEPLER CD	27
10	Drugs & Aging	3.4	Q2	124	HANLON JT	24

Note: N/A indicates the journal is not been included in the list of *Journal Citation Reports*(2023).

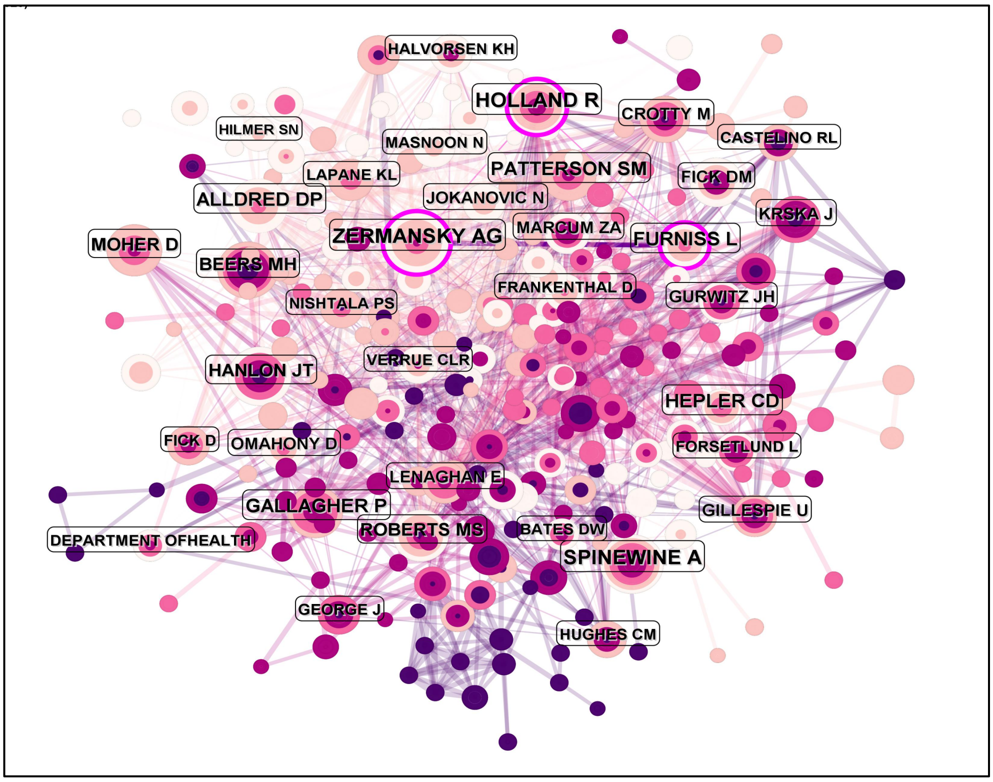

Supplementary Figure 1. The network of author co-citation of home pharmaceutical care

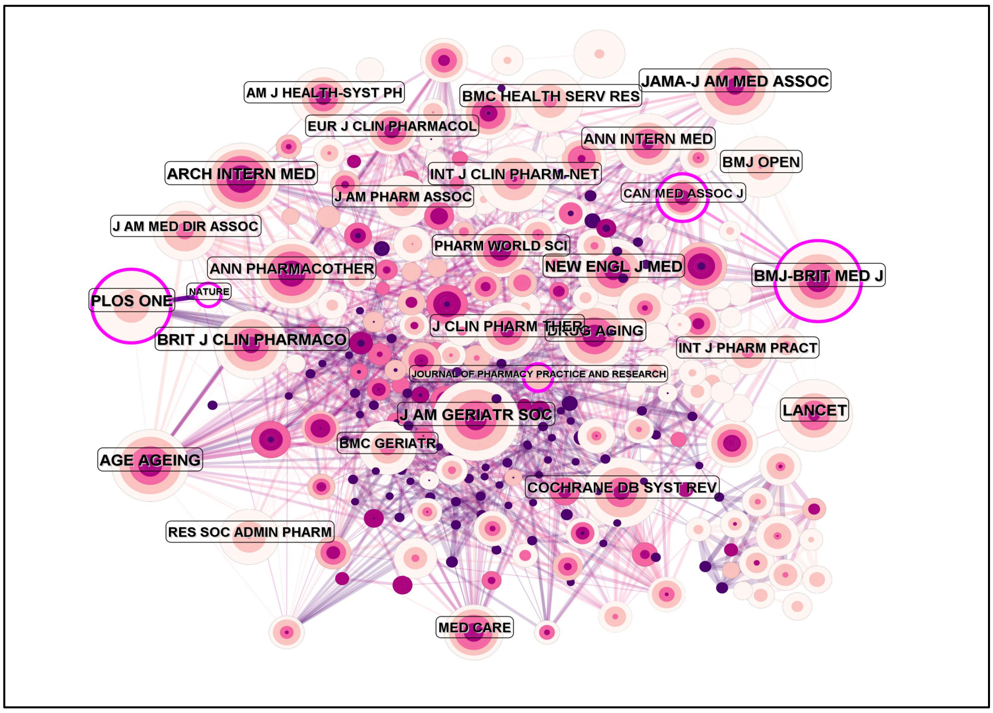

Supplementary Figure 2. The network of journal co-citation of home pharmaceutical care

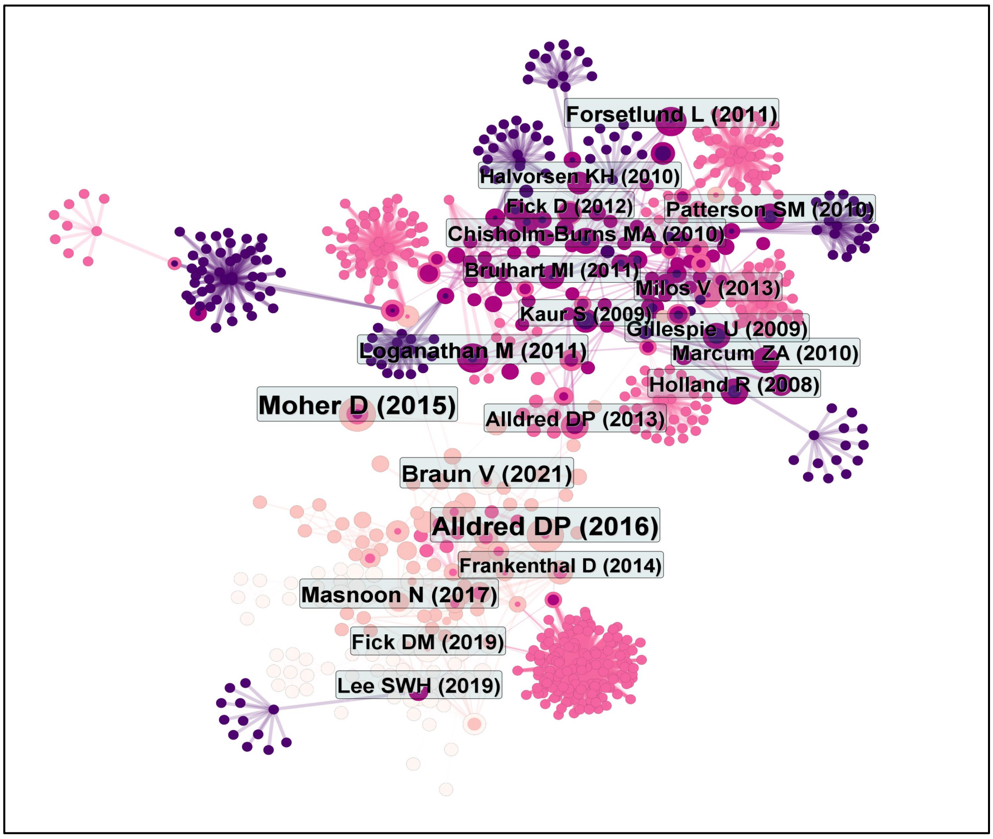

Supplementary Figure 3. The network of co-cited references of home pharmaceutical care

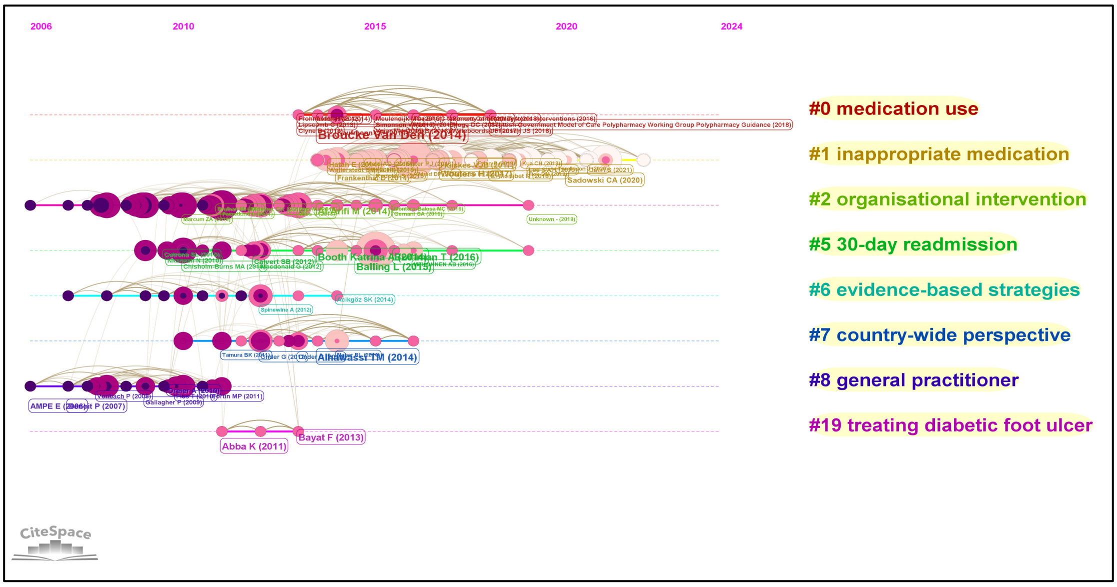

Supplementary Figure 4. The co-cited reference clusters on home pharmaceutical care
